# Utilizing repetitive transcranial magnetic stimulation in the management of gambling disorder in Indonesia: protocol for a pilot and feasibility study

**DOI:** 10.3389/fpsyt.2025.1658195

**Published:** 2025-09-05

**Authors:** Kristiana Siste, Lee Thung Sen, Belinda Julivia Murtani, Enjeline Hanafi, Kevin Surya Kusuma, Astria Aryani, Arnt Schellekens, Philip van Eijndhoven, Iris Dalhuisen, Tom Biemans

**Affiliations:** ^1^ Department of Psychiatry, Faculty of Medicine, Universitas Indonesia, Jakarta, Indonesia; ^2^ Department of Psychiatry, Radboud University Medical Center, Nijmegen, Netherlands; ^3^ Nijmegen Institute for Scientist-Practitioners in Addiction (NISPA), Nijmegen, Netherlands; ^4^ Donders Center for Medical Neuroscience, Donders Institute for Brain, Cognition and Behavior, Nijmegen, Netherlands

**Keywords:** gambling disorder, craving, cognitive-behavioral therapy, repetitive transcranial magnetic stimulation, multimodal therapy

## Abstract

Gambling disorder (GD) has emerged as a significant concern in Indonesia, particularly due to the rise in internet-based gambling during the pandemic and the limited availability of treatment options and support services for affected individuals. This issue has had profound impacts on individuals, their families, and society as a whole. Research has demonstrated that cognitive-behavioral therapy (CBT) is an effective treatment for GD. Additionally, as a relatively novel intervention, several studies have explored the efficacy of repetitive transcranial magnetic stimulation (rTMS) in addiction management, specifically in reducing cravings. This study aims to determine the potential effectiveness and feasibility of combining these two modalities within the Indonesian population. A pilot study of 10 subjects will be conducted to evaluate the effectiveness and feasibility of multimodalistic therapy using rTMS and CBT for GD. South Oaks Gambling Screen (SOGS) will be used to determine the presence of pathological gambling, and the participants will be assessed periodically using SOGS, Gambling Symptoms Assessment Scale (G-SAS), Gambling Urge Scale (GUS), Gambling Related Cognitions Scale (GRCS), Clinical Global Impression (CGI), Self-Reporting Questionnaire-20 Item (SRQ-20), Patient Health Questionnaire-9 Item (PHQ-9), and cognitive assessment. The treatment regimen will consist of 15 sessions of rTMS and 12 sessions of CBT, administered over a 5-week period. The findings of this study will contribute to the development of an optimized rTMS protocol in combination with CBT for the treatment of GD in Indonesia.

**Clinical trial registration:**

ClinicalTrials.gov https://clinicaltrials.gov/study/NCT06598501, identifier NCT06598501.

## Introduction

Gambling disorder (GD), recognized as a behavioral addiction in the Diagnostic and Statistical Manual of Mental Disorders (DSM-5) and the International Classification of Diseases (ICD-11), is a complex and multifaceted condition characterized by persistent and recurrent problematic gambling behavior that leads to significant distress or impairment in daily functioning (1). The prevalence of GD varies globally, influenced by cultural, societal, and regulatory factors (2, 3). Indonesia has faced novel challenges due to the proliferation of online gambling platforms and online lending services during the COVID-19 pandemic, with 2% of the total population suffering from GD (4). This is linear to global prevalence (around 1.29%) with a rising trend observed within the last decade (2, 5). The increasing prevalence of GD in Indonesia is concerning as the lack of available treatment options and support services for individuals affected by GD continues to exacerbate the issue, leading to strained relationships, financial difficulties, and an increased risk of mental health problems such as depression and anxiety (6, 7).

Cognitive-behavioral therapy (CBT) has been demonstrated to be an effective treatment for GD, significantly reducing gambling-related symptoms and problematic behaviors while improving overall functioning and quality of life (8, 9). Another promising modality is repetitive transcranial magnetic stimulation (rTMS), a non-invasive brain stimulation method using electromagnetic induction to modulate brain activity and connectivity (10). Studies using rTMS in the left dorsolateral prefrontal cortex (DLPFC) showed significantly decreased craving symptoms, substance use and compulsive behavior in subjects with nicotine and other substance dependence (11, 12). The Food and Drug Administration (FDA) has approved rTMS as treatment modality for substance dependence but not for behavioral addiction, despite shared clinical features between substance use and gambling disorders. Brain connectivity alterations have also been implicated in GD, with preliminary rTMS data indicating safety and potential effectiveness (13, 14). Study by Hu et al. found a more favorable outcome in alcohol dependent patients who received both CBT and rTMS, acting on the DLPFC neural system to induce neuronal plasticity and expedite neural circuit recovery. Combination of both treatments may also enhance treatment retention and adherence, potentially mitigating the high recurrence rate of GD due to treatment discontinuation (15). Therefore, this study aims to assess the feasibility of the rTMS protocol combined with CBT in Indonesian GD clients.

## Methods

### Study design

This study is a pilot and feasibility study employing a one-arm design. The research protocol adheres to the Standard Protocol Items: Recommendations for Interventional Trials (SPIRIT) checklist (**Supplementary File 1**). After initial screening, participants will undergo complete baseline assessment (T0) and will be given treatment consisting of 15 rTMS sessions, three sessions per week, for a total of 5 weeks. A total of 12 cognitive behavioral therapy (CBT) sessions with psychiatrists will be given at once after baseline assessment, once every two rTMS sessions, and once post-treatment. Each CBT session will last for approximately 60 minutes, which will be divided into 3 phases: (1) preparation, (2) work and (3) summary.

**Figure 1:**
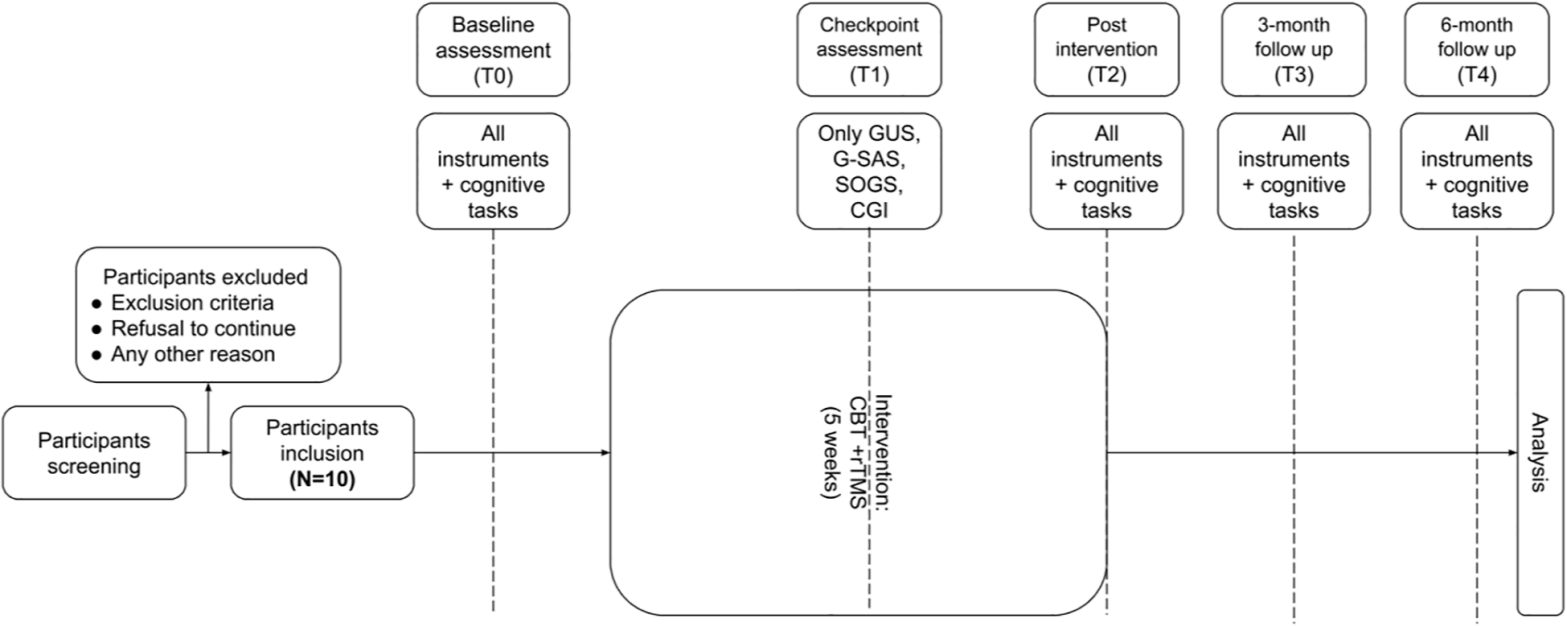
Research Timeline.

Interim assessment during treatment will be done after the seventh rTMS session (T1). This will be followed by a post-treatment assessment (T2) immediately after the last rTMS session, and two follow-up assessments at 3 months (T3) and 6 months (T4) after treatment. For T0, a complete psychiatric interview, cognitive assessment, a demographic questionnaire and 7 instruments will be used, which consist of South Oaks Gambling Screen (SOGS), Gambling Symptom Assessment Scale (G-SAS), Gambling Urge Scale (GUS), Gambling Related Cognition Scale (GRCS), Self-Reporting Questionnaire-20 Item (SRQ-20), Patient Health Questionnaire-9 Item (PHQ-9), and Clinical Global Impression – Severity and Improvement Scale (CGI) (**Table 1**). During T1, SOGS, G-SAS, GUS and CGI will be reassessed and during T2, T3 and T4 all 7 instruments and cognitive assessment will be given to every subject (**Figure 1**). All therapy sessions, as well as assessments at T0, T1, and T2, will be conducted at Cipto Mangunkusumo General Hospital. Assessments at T3 and T4 will be administered either at the hospital or via online platforms to accommodate participants.

**Table 1:** Outcome and measurement.

Outcome	Measure-ment	Data for analysis	Type and score range	Hypothesis	Assessment time point					
					T0	T1	T2	T3	T4	T5
Pathologi- cal gambling	SOGS	Sum of 20 items	Categorical, 0 (no problem with gambling), 1-4 (some problems with gambling), ≥5 (probable pathological gambler).	Lower	v			v		
Gambling symptom severity	G-SAS	Sum of 12 items	Continuous, 0 to 48, with higher scores indicating greater severity of gambling symptoms	Lower	v	v	v	v	v	v
Gambling urges	GUS	Sum of 6 items	Continuous, 0 to 42, with higher scores indicating higher gambling urges/craving	Lower	v	v	v	v	v	v
Gambling- related cognitive distortions	GRCS	5 domains, with the highest percentage being the most dominant domain.	Continuous, 0% to 100% per domain	Lower	v	v	v	v	v	v
Improve- ment of symptoms	CGI	2 domains, severity of illness and global improve-ment	Categorical	Lower	v	v	v	v	v	v
Nonspeci- fic psychologi-cal distress	SRQ-20	Sum of 20 items	Continuous, 0 to 20, with scores >5 indicating mental distress	Lower	v			v		
Degree of depression symptoms	PHQ-9	Sum of 9 items	Categorical, 0 (no depression), 1-4 (minimal depression), 5-9 (mild depression), 10-14 (moderate depression), 15-19 (moderate to severe depression), 20-27 (severe depression)	Lower	v			v		

### Participants and settings

Participants will be recruited through social media platforms, using an online survey to screen potential research subjects. This survey will be administered using the REDCap online survey application (16). The respondents will fill in a demographic questionnaire along with SOGS to assess the presence of pathological gambling. Subjects with pathological gambling (SOGS score ≥ 5) will be contacted by the researcher and explained about the research procedures.

Inclusion criteria will be those: (1) screened with pathological gambling (SOGS score ≥ 5), (2) aged 18–74 years old, (3) comprehend Bahasa Indonesia, (4) consented to participate and receive treatment. Individuals who have (1) history of psychotic disorder and personality disorder according to ICD-11, (2) severe neurological disorder comorbidities, which cause seizure or loss of consciousness, (3) intellectual disability, (4) history of neurostimulation, (5) history of medical implant, (6) currently or expecting pregnancy, (7) fulfilling diagnostic criteria of substance use disorder in the last 6 months will be excluded. Participants may withdraw from the study at any time. Participants will be dropped out from the study if they miss more than two consecutive rTMS sessions. To enhance participant adherence to the treatment regimen, an appointed research associate will be tasked with issuing text message reminders to each participant one day prior to their scheduled treatment. During the study, all participants with previously prescribed medications will be permitted to continue their treatment regimen, with all medications thoroughly screened and closely monitored.

### Development and implementation of the rTMS protocol

The rTMS protocol is developed by the Department of Psychiatry, Faculty of Medicine, Universitas Indonesia, Indonesia and Faculty of Medical Sciences, Radboud University Medical Center, Netherlands. The left LPFC has been shown to be central through functional connectivity in behavioral addiction previously (17). More specifically, alterations in the DLPFC have been implicated in GD through multiple structural and functional imaging studies (18, 19). Response inhibition trials demonstrate decreased activation of the DLPFC and other structures among GD participants (20, 21). In contrast, increased activation was seen during gambling cues and reversal learning tasks (22, 23). Subsequently, enhancing metabolism in the DLPFC had been posited to improve GD symptoms and affect regulation. Similar success was demonstrated in prior transcranial direct current stimulation (tDCS) leading to improved craving and cognitive inhibition (24). Thus, this protocol selects the left DLPFC as the site of stimulation. The protocol parameters are 120% RMT intensity; the stimulation is set to 10 Hz, each train will last for 4 seconds, with 11 seconds of inter-train interval (ITI), and a total of 75 trains thus in total delivering 3,000 waves. Previous rTMS studies have attempted using varying intensity ranging 80-110% and frequency ranging 1-15Hz (24). The frequency 10 Hz was selected. In line with the safety guideline for rTMS, the current protocol capped the intensity at 120% and 4s trains for 10Hz stimulation (25). The rTMS will be delivered using a Neurosoft stimulation system equipped with a 100-mm figure-8 coil (**Supplementary File 2**). The coil will be positioned tangentially to the scalp using a flexible coil holder, with the handle oriented posteriorly and laterally at a 45-degree angle from the midline. This coil orientation allows for targeted stimulation of the DLPFC, a superficial cortical region, while also modulating functionally connected deeper brain structures (26). All rTMS sessions will be conducted by two trained psychiatrists. Treatment will be scheduled by appointment three times per week (Monday, Wednesday, and Friday or Tuesday, Thursday, and Saturday) over a five-week period.

All participants will be screened with a 16-question safety checklist for any contraindications and potential risk factors (i.e. sleep deprivation, metal implants, seizure threshold lowering substances consumption) to rTMS procedure (**Supplementary File 3**) (25). After being declared safe, each participant will have their left DLPFC measured individually using the BeamF3 method (27). This technique involves measuring the nasion-inion distance, tragus-tragus distance, and head circumference to approximate the site of the left DLPFC using a software package, providing a heuristic yet reliable and cost-effective alternative to magnetic resonance imaging-guided neuronavigation. Measurements will be taken with participants wearing a personalized headcap to ensure consistency across sessions (27, 28). Resting motor threshold (RMT) will be identified visually by observing the twitch of the right pollicis brevis muscle (29). Five observable twitches of the muscle in the lowest setting after ten consecutive stimuli will determine the RMT. Prior to each stimulation session, the safety checklist will be re-administered to confirm that no new contraindications or risk factors have emerged. During each session, participants may report any discomfort and after each session they will complete an adverse event assessment. This assessment will focus on pain (including onset, location, quality, duration, and severity), twitching, tingling or redness at the stimulation site and beyond, tinnitus, mood changes, hearing alterations, and other adverse effects (**Supplementary File 4**). Both the safety checklist and adverse event assessment are adapted from Radboud University’s internal assessments checklist and prior guideline for rTMS applications (25).

### Administering the CBT for GD

The CBT for GD module that will be utilized in this study was adapted from Indonesia Drug Addiction and Relapse Prevention Program (Indo-DARPP) CBT module for substance use disorders, with modifications based on established gambling-specific CBT frameworks (30–34).The CBT module for this study consists of 12 sessions, each lasting approximately 30–45 minutes. Sessions will be held individually in person and delivered once for every two rTMS session, depending on participant availability. The CBT module addresses a range of topics relevant to GD, including: (1) the definition, symptoms, and underlying mechanisms of GD; (2) individualized gambling-related harms; (3) reasons to stop gambling; (4) stages of change; (5) gambling triggers; (6) the gambling cycle; (7) gambling craving and extinction strategies; (8) development of alternative, non-gambling routines; (9) financial management skills; (10) rebuilding interpersonal relationships affected by gambling behavior; (11) emotional regulation strategies; and (12) relapse prevention planning.

### Instruments for study outcomes

This study will employ four instruments to assess gambling related outcomes (SOGS, G-SAS, GUS, GRCS), two instruments to evaluate mental health symptoms (PHQ-9 and SRQ-20), and one clinician-rated instrument to assess symptom severity and improvement over the course of treatment (CGI). The selected gambling-related instruments have demonstrated strong reliability and validity. These instruments are widely used internationally and available in Bahasa Indonesia to capture various dimensions of gambling behavior (35–38). Specifically, the SOGS will be used not only to screen for pathological gambling but also to gather information on participants’ preferred gambling activities, the presence of gambling culture within their family or social environment, and sources of gambling funds (35). The G-SAS will assess fluctuations in gambling symptoms severity, while the GUS will measure gambling cravings experienced throughout the study (36, 37). The GRCS will identify cognitive distortions related to gambling among participants (38). Regarding mental health outcomes, the PHQ-9 will assess depressive symptoms, and the SRQ-20 will evaluate general psychological distress (39, 40). The CGI will provide insights to progress made by the participants based on clinical judgment (41).

### Participant characteristics

The subsequent demographic information will be gathered through a self-administered questionnaire: age, sex, ethnicity, educational level, marital status, employment status, monthly earnings, most recent gambling experience, age of initial gambling experience, frequency of gambling per month, initial exposure to gambling, presence of other individuals with gambling issues in the patient’s circle, preferred gambling platforms, types of devices used, varieties of gambling engaged in, typical duration of gambling on weekdays and weekends, minimum and maximum wagers ever placed, total monetary losses from gambling, motives behind gambling, negative consequences from gambling, attempts to seek help, history of substance use, substance consumption during gambling, and, if applicable, experience of rehabilitation programs due to substance use.

### Sample size

Based on convention in previous safety studies of medical interventions, the study sample ranged below 20 subjects (Murshed, 2019) (42). A study assessing pre-screening rTMS questionnaires in adults previously used 15 subjects (Keel et al., 2001) while a European feasibility study used 10 subjects (43, 44). Therefore, the current feasibility study will also use a sample size of 10 subjects.

Because this study is a pilot and feasibility study, we used references from previous studies related to the potential of rTMS as a therapy for gambling addiction. However, previous research has not examined the efficacy and combination of therapy, and no research has succeeded in determining standards for these variables. Therefore, we refer to studies of the safety and feasibility of rTMS.

### Statistical analysis

Statistical analysis will be conducted to evaluate the treatment response using generalized linear mixed model. The Statistical Package for the Social Sciences (SPSS) software version 25.0 will be used for this analysis. The raw data obtained in this study only be accessible to the authors. Subgroup analysis will not be conducted in this study.

### Data monitoring

Collected data within the study encompass socio-demographic information, psychometric assessments, and rTMS measurements, all of which will be securely stored and accessible solely to the research team. This study does not involve the collection of biological specimens. Participants will undergo assessments both before and after each rTMS session to monitor any adverse effects (such as headaches, fatigue, mood alterations, etc.) resulting from the intervention. Should any adverse effects be noted, participants will be promptly referred to appropriate medical professionals for comprehensive evaluation and treatment. Any subsequent medical expenses not already covered will be fully provided for by the investigators.

## Discussion

This will be the first study to examine the effectiveness and feasibility of rTMS treatment modality for GD in Indonesia. GD therapeutic options are still limited, with no pharmacotherapy proven effective. Opioid antagonists have been suggested as potential therapy, but the results were inconclusive (45). Although CBT is widely used for treating GD with effectiveness of improving gambling related symptoms and overall quality of life, the improvements may not be immediate and often require months to show its desired effects (46). rTMS treatment modality has the added benefit of neuromodulation and inducing neuroplasticity in the targeted brain region (14). A decrease of striatal dopamine transporter (DAT) availability is observed in individuals with GD compared to healthy controls. This reduced availability of dopamine was found to be inversely correlated with gambling frequency and reward-based decision-making in individuals with GD (47). Thus, restoring dopamine function is hypothesized to benefit addiction treatment (48).

A study by Strafella et al. discovered that rTMS targeted to the left mid-dorsolateral prefrontal cortex was able to induce the release of dopamine in the striatal region, potentially benefiting many clinical aspects (49). Reduced cognitive control in GD may be linked to abnormal activity in the DLPFC, anterior cingulate cortex (ACC), and orbitofrontal cortex (OFC). Impaired DLPFC function is associated with deficits in working memory, which affects decision-making in GD (50). Stimulation using rTMS targeting glutamatergic and dopaminergic systems through the DLPFC showed potential for treatment (44). Previous rTMS studies have targeted both the right and left DLPFC, with improvements in craving and cognitive control observed in studies targeting the left rather than right DLPFC, suggesting a preference for the left DLPFC. Furthermore, high frequency rTMS is also preferred as several studies using high frequency rTMS showed notable improvements while low frequency rTMS reported no significant changes (14).

These findings do not undermine CBT as the preferred therapy for GD but emphasize the potential advantages of a multimodal treatment approach. Combining CBT with other modalities, such as pharmacotherapy and neurostimulation, may improve treatment outcomes for GD clients.
