# Supplementary material for: Utilizing repetitive transcranial magnetic stimulation in the management of gambling disorder in Indonesia: protocol for a pilot and feasibility study
Source: Front Psychiatry. 2025 Sep 5;16:1658195. doi: 10.3389/fpsyt.2025.1658195 (PMC12447642; doi:10.3389/fpsyt.2025.1658195)
Supplement: Supplementary file 2 [file Supplementaryfile2.docx]

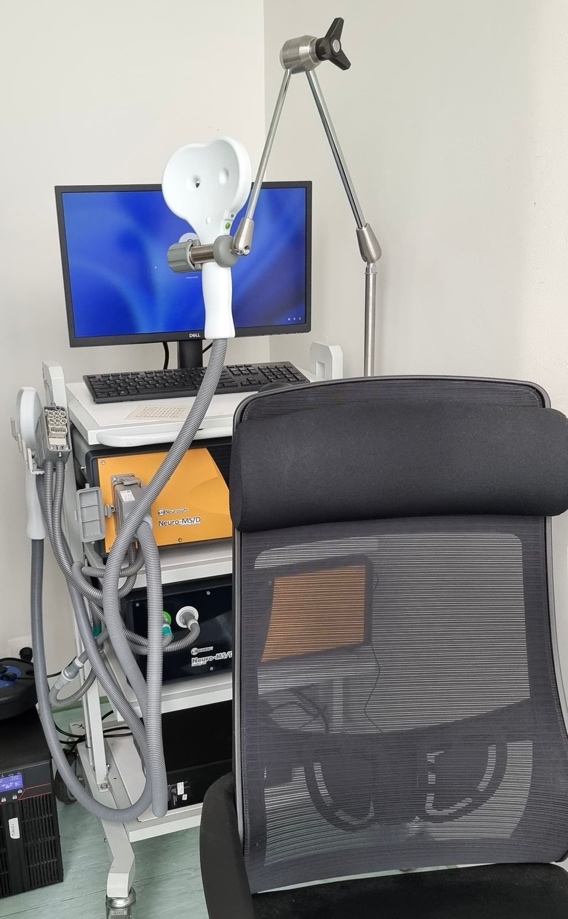


Picture A. front view of rTMS device used in this study (only one coil is necessary)


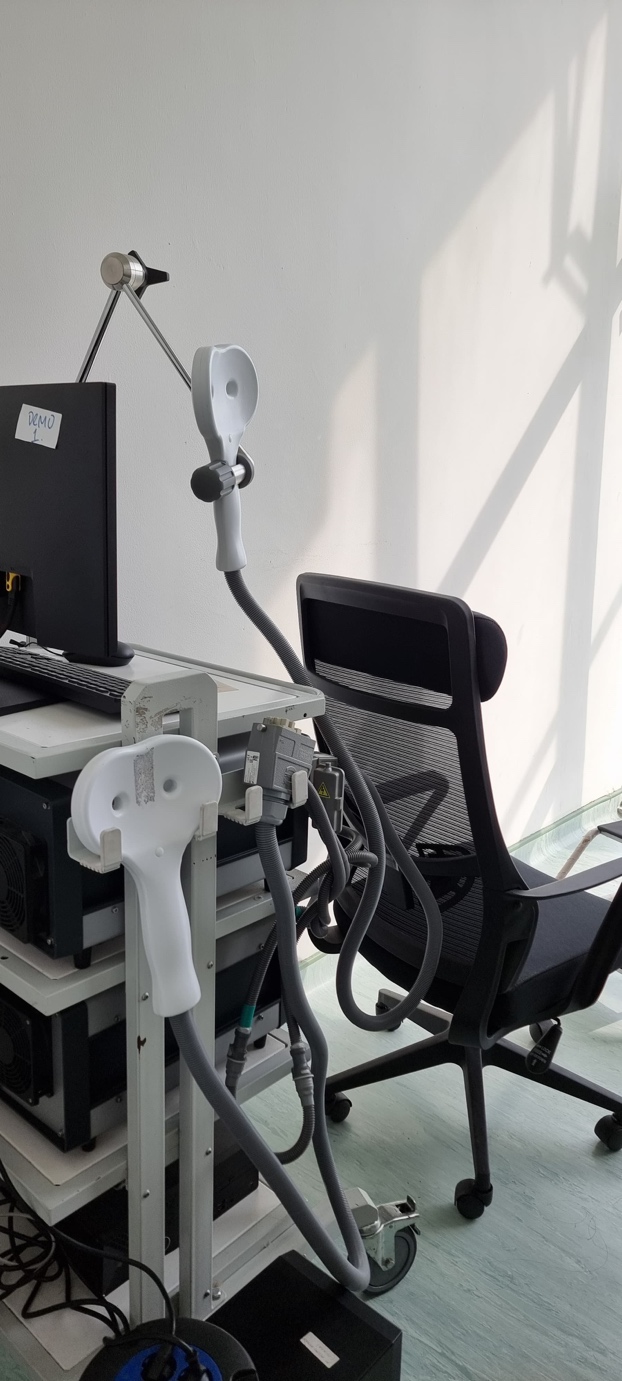


Picture B. Side view of rTMS device used in this study (only one coil is necessary)
