# Supplementary material for: Utilizing repetitive transcranial magnetic stimulation in the management of gambling disorder in Indonesia: protocol for a pilot and feasibility study
Source: Front Psychiatry. 2025 Sep 5;16:1658195. doi: 10.3389/fpsyt.2025.1658195 (PMC12447642; doi:10.3389/fpsyt.2025.1658195)
Supplement: Supplementary file 3 [file Supplementaryfile3.docx]

Week 1 / 2 / 3 /4/5/ 6 Session 1 / 2 / 3 /4/ 5

rTMS Screening Questionnaire for each TMS session

Tick ​​the next question and the answer that most applies is Yes or No

| No | Questions | Yes | No | Comments |
| --- | --- | --- | --- | --- |
| 1. | Has anything changed in your health since your last rTMS session? |  |  |  |
| 2. | Do you notice that you suffer from:  a) headache  b) dizziness  c) scalp problems  d) something else, …………………. |  |  |  |
| 3. | Have you been to the doctor/hospital since the last TMS session (eg for an epileptic seizure)? |  |  |  |
| 4. | Have you used (recreational) drugs since the last session (benzodiazepines, anti-allergy drugs, ecstasy)? |  |  |  |
| 5. | Has anything changed in the dose of your current medications? If yes, please specify: |  |  |  |
| 6. | Have you consumed alcohol in the past 24 hours? If so, how much? |  |  |  |
| 7. | Have you consumed caffeine in the past 24 hours? If so, how much? |  |  |  |
| 8. | Did you get a normal amount of sleep last night? If not, how many hours? |  |  |  |
| 9. | Have any side effects related to the rTMS occurred after or during the last treatment, if yes go to side effects questionnaire. |  |  |  |
| 10. | Do you have any hearing problems or ringing in your ears? |  |  |  |
| 11. | Do you have cochlear implants? |  |  |  |
| 12. | Are you pregnant or is there any chance that you might be? |  |  |  |
| 13. | Do you have metal in the brain, skull or elsewhere in your body (e.g., splinters, fragments, clips, etc.)? If so, specify the type of metal. |  |  |  |
| 14. | Do you have an implanted neurostimulator (e.g., DBS, epidural/subdural, VNS)? |  |  |  |
| 15. | Do you have a cardiac pacemaker or intracardiac lines? |  |  |  |
| 16. | Do you have a medication infusion device? |  |  |  |
| 17. | Are you taking any medications? |  |  |  |

Participant

Name : Date:

Researcher

Name : Date:

Signature :

Radboud University. rTMS Screening Questionnaires. Nijmegen: Radboud University
