# Supplementary material for: Utilizing repetitive transcranial magnetic stimulation in the management of gambling disorder in Indonesia: protocol for a pilot and feasibility study
Source: Front Psychiatry. 2025 Sep 5;16:1658195. doi: 10.3389/fpsyt.2025.1658195 (PMC12447642; doi:10.3389/fpsyt.2025.1658195)
Supplement: Supplementary file 4 [file Supplementaryfile4.docx]

|  | When did the sensation begin | | | | How long did sensation last | | | | Location | |
| --- | --- | --- | --- | --- | --- | --- | --- | --- | --- | --- |
|  | After the start of the session | In the middle | At the end | After the session | Quickly stopped | Stopped mid-session | At the end of the session | After the session | Diffuse | At the stimulation site |
| Have you suffered from | 1/2/3/4 | 1/2/3/4 | 1/2/3/4 | 1/2/3/4 | Time in minutes | Time in minutes | Time in minutes | Time in minutes |  |  |
| Skull pain |  |  |  |  |  |  |  |  |  |  |
| Sore throat |  |  |  |  |  |  |  |  |  |  |
| Tingling sensation skull |  |  |  |  |  |  |  |  |  |  |
| Tingling sensation in peripheral nerves |  |  |  |  |  |  |  |  |  |  |
| Itch |  |  |  |  |  |  |  |  |  |  |
| Tingling sensation in peripheral nerves |  |  |  |  |  |  |  |  |  |  |
| Headache |  |  |  |  |  |  |  |  |  |  |
| Tinnitus |  |  |  |  |  |  |  |  |  |  |
| Skin redness or skin sensations |  |  |  |  |  |  |  |  |  |  |
| Twitching (except slap) |  |  |  |  |  |  |  |  |  |  |
| Fatigue |  |  |  |  |  |  |  |  |  |  |
| Drowsiness |  |  |  |  |  |  |  |  |  |  |
| Changes in hearing |  |  |  |  |  |  |  |  |  |  |
| Changes in mood (depression) |  |  |  |  |  |  |  |  |  |  |
| Changes in mood (euphoria) |  |  |  |  |  |  |  |  |  |  |
| Nausea |  |  |  |  |  |  |  |  |  |  |
| Neck stiff/ neck pain |  |  |  |  |  |  |  |  |  |  |
| Fear/nervousness |  |  |  |  |  |  |  |  |  |  |
| Difficulty in concentration |  |  |  |  |  |  |  |  |  |  |
| Other |  |  |  |  |  |  |  |  |  |  |

Radboud University. rTMS side effects questionnaires. Nijmegen: Radboud University
